# Synchronous Appearance of a High-Grade Neuroendocrine Carcinoma of the Ampulla Vater and Sigmoid Colon Adenocarcinoma

**DOI:** 10.1155/2013/930359

**Published:** 2013-12-03

**Authors:** Suna Cokmert, Lutfiye Demir, Aysegul Akder Sari, Yuksel Kucukzeybek, Alper Can, Murat Akyol, Ibrahim Vedat Bayoglu, Ahmet Dirican, Cigdem Erten, Mustafa Oktay Tarhan

**Affiliations:** ^1^Department of Medical Oncology, Izmir Ataturk Training and Research Hospital, Izmir Katip Celebi University, 35600 Izmir, Turkey; ^2^Department of Pathology, Izmir Ataturk Training and Research Hospital, Izmir Katip Celebi University, 35600 Izmir, Turkey

## Abstract

Neuroendocrine carcinoma is a relatively rare tumor and its coexistence with other primary cancers is very exceptional. We present a case of a 63-year-old woman with biliary obstruction due to a high-grade neuroendocrine carcinoma located in ampulla of Vater who was found to have a synchronous sigmoid colon adenocarcinoma while undergoing staging laparotomy and pancreas head resection. Medical history was significant only for basal cell skin cancer. Immunohistochemical examination revealed the concurrence of histologically proved neuroendocrine carcinoma (chromogranin A, synaptophysin, and CD56 were positive) and Stage II (T3, N0, and M0) according to the TNM staging classification of colorectal cancer. The coexistence of neuroendocrine tumors with either synchronous or metachronous unrelated cancer is increasingly recognized. The patients with neuroendocrine carcinoma should be evaluated for secondary primary malignancies.

## 1. Introduction 

Neuroendocrine tumors (NETs) are a rare and heterogeneous group of neoplasms [1] that can arise from neuroendocrine cells localized anywhere in the body [1, 2]. In the duodenum, NETs constitute 5.7 to 7.9% of the neuroendocrine neoplasms of the gastroenteropancreatic tract [3]. In the English language literature, fewer than 120 cases of NETs of the ampulla of Vater have been described. Furthermore, most neuroendocrine tumors of the duodenum are of carcinoid origin and only a few case reports of neuroendocrine carcinomas (NEC) of the ampulla have been documented [4–10].

Neuroendocrine tumors are associated with synchronous or metachronous secondary primary malignancies. Rates of secondary primary malignancies are up to 55% in neuroendocrine tumors [11]. Secondary primary malignancies are mainly localized in the gastrointestinal and genitourinary tract. Neuroendocrine carcinoma of the ampulla of Vater very rarely coexist with other primary sporadic cancers. Here, we present a patient with an ampulla of Vater mass, diagnosed as a high-grade neuroendocrine carcinoma, and a synchronous sigmoid colon adenocarcinoma coincidentally diagnosed during the operation for the ampulla of Vater tumor.

## 2. Case Report

**Figure 1:**
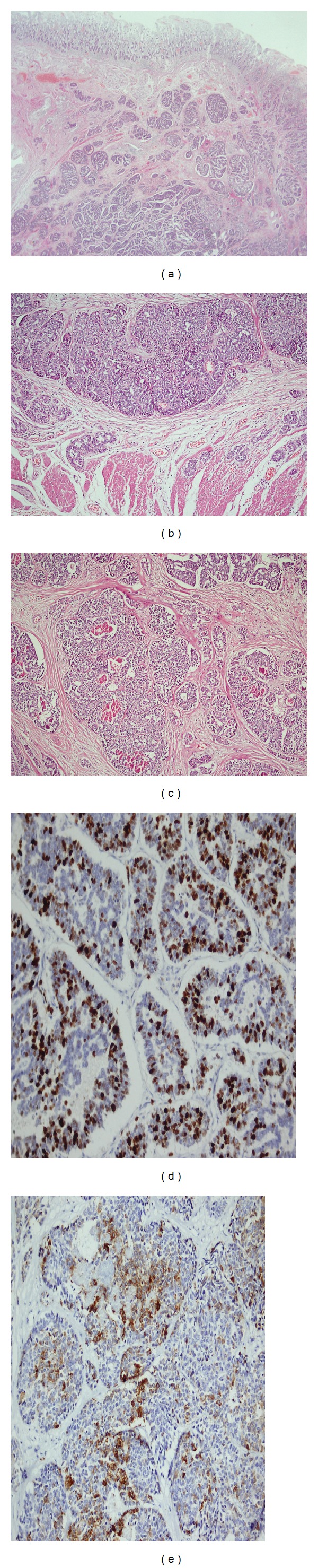
Neuroendocrine carcinoma of ampulla of Vater. (a)-(b) Neuroendocrine tumor invading muscularis propria in the ampulla, (c) focal necrosis centres and moderate atypia in the tumor, (d) Ki-67 proliferation index above 20%, (e) focal positivity with synaptophysin.

**Figure 2:**
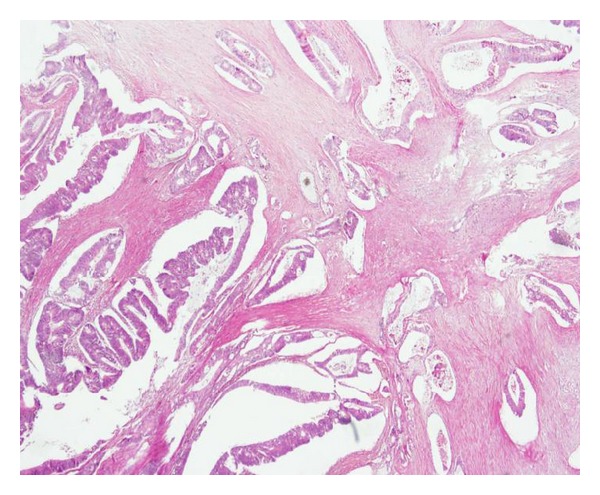
Adenocarcinoma in sigmoid colon.

A 63-year-old woman presented with a one-month history of progressive upper abdominal pain and jaundice. On physical examination both sclerae appeared yellow. Abdominal examination revealed mild tenderness in the right upper abdominal quadrant, but no mass was palpable. There were no changes in her bowel movements. Medical history was insignificant except for basal cell skin cancer for which she had undergone surgery sixteen years ago. Blood tests showed mildly impaired liver function tests (total bilirubin: 1.9 (0-1 mg/dL), direct bilirubin: 1.1 (0–0.3 mg/dL), AST: 22 (0–32 IU/L), ALT: 18 (0–32 IU/L), ALP: 189 (40–129 IU/L), and GGT: 44 (10–60 U/L)). Hemogram results revealed microcytic, hypochromic anemia, with hematocrit 24.2% (reference range 36–46%), hemoglobin 7.65 g/dL (reference range 12–18 g/dL), mean corpuscular volume 69.2 fL (reference range 80–97 fL), mean corpuscular hemoglobin 22 pg (reference range 27–31 pg), mean corpuscular hemoglobin concentration 31.8 g/dL (reference range 32–36 g/dL), and serum ferrum and ferritin levels of 23 *μ*g/dL (reference range 70–180 *μ*g/dL) and 80 *μ*g/L (reference range 30–400 *μ*g/L), respectively. The tumor markers alpha-fetoprotein (AFP), carcinoembryonic antigen (CEA), and CA19-9 were all within normal ranges. Ultrasonographic examination of the abdomen showed a dilation of choledocal duct but no gallstones. Abdominal computed tomography showed no enlargement of the pancreatic head. An endoscopic retrograde cholangiography revealed a mass in the ampulla of Vater. Endoscopic biopsy was taken from mass lesion and showed a poorly differentiated neuroendocrine carcinoma. As the patient had no hormone-related symptoms, octreoscan was not used and a decision was made to perform resection. A pancreatoduodenectomy was done. During surgery, a mass in the sigmoid colon was incidentally found. Left colectomy and a lymphadenectomy were performed. Histopathological examination showed a poorly differentiated neuroendocrine carcinoma of ampulla of Vater (Figures 1(a)–1(c)), 1.2 cm in diameter, with a mitotic rate of nine mitoses per 10 high power fields and a Ki-67 proliferative index of 40% (Figure 1(d)). Immunohistochemistry chromogranin A, synaptophysin (Figure 1(e)), and CD56 were positive. There was no metastasis to periampullary lymph nodes. Furthermore, there was a sigmoid colon adenocarcinoma with local invasion into the subserosa (pT3) (Figure 2). None of the 8 resected pericolic lymph nodes were involved. All microscopic margins were clear. Treatment with 5-FU and leucovorin plus cisplatin to treat both tumors was suggested postoperatively.

## 3. Discussion

Neuroendocrine tumors (NETs) may be localized throughout the human body [1] and relatively rarely in theduodenum [2, 3]. In an analysis of 13,715 neuroendocrine tumors reported over a 50-year period to the Surveillance, Epidemiology, and End Results Program of the National Cancer Institute, only 360 cases involved the duodenum or ampulla [9, 12]. A series from Memorial Sloan-Kettering Cancer Center showed only 14 high-grade neuroendocrine carcinomas out of 215 ampullary carcinomas [13]. Carter et al. reported 7 patients with NET of ampulla of Vater and, among those patients, a high-grade neuroendocrine carcinoma was detected in only one patient [9]. Based on these reports, it can be postulated that high-grade neuroendocrine carcinoma localized in ampulla of Vater is very rare.

Neuroendocrine tumors are associated with secondary primary malignancies. In 1944, Pearson and Fitzgerald reported, for the first time, a high incidence of carcinoid tumors with secondary primary malignancies (SPM) [14]. Many reports demonstrated NET-associated SPM in up to 55% [3–10] of patients. The situation of NET-associated SPM can be explained with the field-effect theory. Accordingly, a common carcinogenic effect stimulates the growth of neuroendocrine and SPM cancer cells [11, 15]. Additionally, NETs produce and secrete various neuropeptides or nonneuropeptides, many of which have specific growth factor properties [16]. For example, gastrin and cholecystokinin (CCK) can stimulate gastric mucosal and pancreatic cell growth [17]. Recently, receptors for CCK and gastrin were detected in large amounts in the tissues of lung, ovarian, thyroid, and brain tumors [18]. Although there are several reports on the increased risk for a second primary malignancy (SPM) in patients with carcinoid tumors [15, 17, 19], the rate of secondary primary malignancies for high-grade neuroendocrine carcinoma is unknown. In our patient, there was a high-grade neuroendocrine carcinoma located in the ampulla of Vater. Her colon tumor was asymptomatic and her abdominal CT showed no abnormal finding, and the mass in the sigmoid colon was coincidentally found during surgery. Synchronous double primary tumors involving high-grade neuroendocrine carcinoma of ampulla of Vater and sigmoid colon adenocarcinomas have not been reported in the literature, making this the first case report of this scenario. Prommegger et al. reviewed 14 patients with NET and SPM, and, among those patients, a NET of duodenum localization was detected in only two patients whose SPM were basal cell carcinoma of skin and colon carcinoma. Five patients had synchronous SPM including two colon cancers with one double colon cancer, one gastric cancer, one bladder cancer, and one ovarian cancer and nine metachronous SPM including two basal cell carcinomas, one colon cancer, two breast cancer, one gastric MALT-lymphomas, one ductal pancreatic adenocarcinoma, one bladder cancer, and one hepatocellular carcinoma [20]. In addition, a case was reported of a woman treated initially for a synchronous squamous cell carcinoma of the cervix and a basal cell carcinoma of the skin, who developed a third malignancy described as a neuroendocrine carcinoma of an unknown primary site [21]. Our patient has been diagnosed with basal cell skin carcinoma 15 years prior to the initial diagnosis of the synchronously described colon adenocarcinoma and neuroendocrine carcinoma.

In this report, we present an interesting case with high-grade neuroendocrine carcinoma of ampulla of Vater and asymptomatic synchronous sigmoid colon adenocarcinoma incidentally detected at the operation. Multiple primary tumors seen synchronously and/or metachronously, with neuroendocrine carcinomas, are an increasingly encountered phenomenon. These patients must be extensively evaluated for SPM during the workup and follow-up period. We recommend that a whole body CT scan and endoscopic investigation of the gastrointestinal tract be performed. Further studies are required to clarify the mechanisms of carcinogenesis associated with neuroendocrine carcinomas and synchronous tumors.
